# Implementation strategies in emergency management of children: a scoping review protocol

**DOI:** 10.1186/s13643-020-01310-z

**Published:** 2020-03-03

**Authors:** Alex Aregbesola, Ahmed M. Abou-Setta, Maya M. Jeyaraman, George Okoli, Otto Lam, Kathryn M. Sibley, Terry P. Klassen

**Affiliations:** 1 The Children’s Hospital Research Institute of Manitoba, John Buhler Research Centre, 513-715 McDermot Avenue, Winnipeg, MB R3E 3P4 Canada; 2 Department of Pediatrics and Child Health, Max Rady College of Medicine, Rady Faculty of Health Sciences, University of Manitoba, Winnipeg, MB Canada; 3 George & Fay Yee Centre for Healthcare Innovation, Max Rady College of Medicine, Rady Faculty of Health Sciences, University of Manitoba, Winnipeg, MB Canada; 4 Department of Community Health Sciences, Max Rady College of Medicine, Rady Faculty of Health Sciences, University of Manitoba, Winnipeg, MB Canada

**Keywords:** Implementation strategies, Pediatrics, Emergency, Emergency department, Emergency management, Scoping review

## Abstract

**Background:**

Behavior change is not simple, and the introduction of guidelines or protocols does not mean that they will be followed. As such, implementation strategies are vital for the uptake and sustainability of changes in medical protocols. Medical or mental emergencies may be life-threatening, especially in children due to their unique physiological needs. In emergency departments (EDs), where timely decisions are often made, practice change requires thoughtful considerations regarding the best approaches to implementation. As there are many studies reporting on a wide variety of implementation strategies in the emergency management of children in EDs, we aim to identify and map the characteristics of these studies.

**Methods:**

We will conduct a scoping review to identify various implementation strategies in the emergency management of children using the Arksey and O’Malley framework. We will search MEDLINE (Ovid), Embase (Ovid), Cochrane Central (Wiley), and CINAHL (Ebsco), from inception to May 29, 2019, for implementation studies among the pediatric population (≤ 21 years) in a pediatric emergency setting. Two pairs of reviewers will independently select studies for inclusion and extract the data. We will perform a descriptive, narrative analysis of the characteristics of the identified implementation strategies.

**Discussion:**

We will present specific characteristics and outcome measures of all included studies in a tabular form. The results of this review are expected to help identify and characterize successful implementation strategies in the emergency management of children in EDs.

**Systematic review registration:**

Open Science Framework https://osf.io/h6jv2

## Background

It is well documented that a delay in response to medical emergencies may lead to fatal outcomes, which may be heightened in children due to their unique physical and psychosocial needs [1]. Likewise, the requirements to manage pediatric emergencies differ from adults because of their unique needs in medication, equipment, staff, and pediatric-specific policies and protocols [2]. Children are often vulnerable to receiving treatments based on adult guidelines because a significant percentage of pediatric emergencies is managed in the adult or general emergency department (ED) [3]. Thus, there is a need to identify implementation strategies that can help promote guidelines or protocols targeted towards this subpopulation in emergency settings. There is evidence that the quality of care in pediatric emergencies has improved with the implementation of evidence-based guidelines in the emergency management of children [4, 5]. To continue this trend, and implement it in other areas of care, we first must identify successful implementation strategies and identify patterns of success among these strategies.

In general, implementation strategies are methods or techniques used to enhance the uptake and sustainability of a program or practice [6]. They can be categorized into the following classes: (1) dissemination strategies (including developing messages and materials, distribution of evidence-based information), (2) implementation process strategies, (3) integration strategies, and (4) capacity building and scale-up strategies [7]. These strategies can interact with one another within a framework, or be used independently, to promote research uptake [7].

Successful implementation strategies in this context, therefore, are strategies that lead to an increase in the uptake or utilization of guidelines or protocols into routine practice [8]. Although the preferred outcome in implementation research is to promote the overall quality of healthcare, the success of implementation strategies is in being able to positively influence healthcare professional and organization behavior to accept or utilize evidence-based practices [8].

While some recent studies examining the effect of implementing guidelines in emergency management of children have found positive outcomes [4, 5, 9], others have reported no significant effects on the outcomes [10]. It is unclear if this is a result of poor implementation strategies or lack of reporting on what entailed the implementation strategies. For example, Corwin et al. [9] examined the effect of implementing the Pediatric Emergency Care Applied Research Network (designed to reduce unnecessary neuroimaging in children presenting with mild traumatic brain injury) on the use of computed tomography (CT) scan in pediatric ED. They found a decrease in the rate of head CT scan use after the guideline was implemented using provider feedback and electronic decision support to prompt health providers to adopt the pathway. And as such, they adopted integration strategies, which include instituting reminder systems to improve the uptake of guidelines [7]. A before and after study [10], which examined the effect of the implementation of a pain guideline in the pediatric ED, found that the pain protocol did not reduce time to analgesia administration. They utilized capacity building and scale-up strategies, which include training the ED physicians and nurses before the implementation of the guideline [7].

To better understand the characteristics of successful implementation strategies in EDs that attend to children, the aim of this scoping review is to identify the various implementation strategies and characterize the successful ones in the emergency management of children.

## Methods

We will use a multidisciplinary team, with expertise in emergency management of children, pediatrics, research methodology, and implementation science, to identify evidence to answer our review question: What are the characteristics of successful implementation strategies used in emergency management of children?

We will adopt Arksey and O’Malley’s 5-stage framework to conduct the scoping review [11], by identifying and stating our research questions, eligibility criteria, search strategy, and study selection; charting included data; and collating and summarizing our results. The present protocol has been registered within the Open Science Framework platform (registration ID: https://osf.io/h6jv2) and is being reported in accordance with the reporting guidance provided in the Preferred Reporting Items for Systematic Reviews and Meta-Analyses Protocols (PRISMA-P) statement [12] (see checklist in Additional file 1).

### Study eligibility criteria

This review will focus on studies conducted in an emergency management setting, reporting evidence used on individuals expected to be under pediatric care (e.g., 21 years and below [13], who were managed in an emergency setting). Our focus is on controlled studies that applied a protocol/guideline or a specific treatment or treatment plan in an emergency setting compared to before implementation or to another setting in which the implementation strategy was not applied. Our intervention of interest will be any implementation strategies as described earlier [7]. The literature will be limited to peer-reviewed, full-text articles published in English. There will also be no limits on the date of publication. We will exclude studies that do not mention any implementation strategy in the application of protocols/guidelines/treatment/treatment plans in the management of pediatric emergencies.

### Search strategy

A medical librarian has designed and will execute a literature search strategy in MEDLINE (Ovid) from inception through May 29, 2019 (see Additional file 2). The search strategy will also then be adapted for other bibliographic databases: Embase (Ovid), Cochrane Central (Wiley), and Cinahl (Ebsco). All retrieved records will be imported into an Endnote (X8).

### Study selection

Two pairs of reviewers will independently screen the identified citations for eligibility using a two-stage sifting approach to review the title, abstract, and full-text article. We will record the number of ineligible citations at the title and abstract screening stage, and both the number and reason for ineligibility at the full-text articles. Disagreements will be resolved by a discussion between reviewers or by involving another reviewer when necessary.

### Data extraction

We will develop data extraction forms in MS Excel (Microsoft Corporation, Redmond, WA, USA) and pilot them on a small selection of studies. For each included study, data will be extracted by one reviewer and checked by another for errors. Disagreements will be resolved by discussion between reviewers or by involving another reviewer when necessary. We will extract the following data:

1. Study details: first author, year of publication, country, study design, study period, study objective (what is being implemented), and area of study (classification)
2. Intervention: use of any of the implementation strategies, dissemination strategies (including developing messages and materials, distribution of evidence-based information), implementation process strategies, integration strategies, capacity building and scale-up strategies, and the numbers and types of implementation strategies used

## Results

- Number/proportion of participants after the intervention implementation, effect estimate measured (e.g., percentage/proportion/mean difference/odds ratio/hazards ratio/relative risk/risk difference). This is a direct effect on providers.
- Number/proportion of patients receiving intervention after implementation, effect estimate measured (e.g., percentage/proportion/mean difference/odds ratio/hazards ratio/relative risk/risk difference). This is an indirect effect on providers.

### Data analysis

We will present specific characteristics and outcome measures of all included studies in a tabular form. The analysis of the extracted data will be descriptive. A summary of different types of implementation strategies in the emergency management of children, the types of study designs, and the direct and/or indirect effects produced will be presented in a narrative format.

## Discussion

To the best of our knowledge, this will be the first systematic scoping review identifying the implementation strategies in the emergency management of children. This scoping review will provide an evidence base map of various implementation strategies that have been used in the emergency management of children and, more importantly, characterize successful implementation strategies. Following our preliminary literature search on this study question, although we expect a significant number of studies to meet our inclusion criteria, we also anticipate diversities in the implementation strategies used in the emergency management of children.

This review will follow standard accepted methods for scoping reviews and will be reported according to the new PRISMA guidelines. In addition, the inclusion of an experienced systematic review team, including an expert in implementation science, will provide adequate guidance to the reviewers during study selection, data extraction, and interpretation of the results. Even so, while the search strategy was clearly defined and relatively extensive, we anticipate some limitations in the scoping review to capture all the available studies related to the emergency management of children. Because of our inclusion criteria, studies may be omitted if not indexed in the databases we searched, full-text not available, or if reported in other languages other than English.

Taken together, the scoping review will help to identify successful implementation strategies in the emergency management of children, which will help to prioritize approaches and measures while implementing protocols or guidelines in pediatric emergency settings.

## Knowledge dissemination strategy

We will submit reports from this study for peer-reviewed publication in appropriate academic journals. There will also be a presentation of our findings at provincial, national, and international scientific meetings/conferences.

## Supplementary information

**Additional file 1.** PRISMA Checklist.

**Additional file 2.** Literature search strategy in MEDLINE (Ovid).

## Data Availability

Not applicable.

## Abbreviations

CT: Computed tomography
ED: Emergency department
PRISMA: Preferred Reporting Items for Systematic Reviews and Meta-Analyses
