# Supplementary material for: Implementation strategies in emergency management of children: a scoping review protocol
Source: Syst Rev. 2020 Mar 3;9:46. doi: 10.1186/s13643-020-01310-z (PMC7055076; doi:10.1186/s13643-020-01310-z)
Supplement: Supplementary file 2 — Additional file 2. Literature search strategy in MEDLINE (Ovid). [file 13643_2020_1310_MOESM2_ESM.docx]

**Appendix A**: Literature Search Strategy

Database: Ovid MEDLINE(R) and Epub Ahead of Print, In-Process & Other Non-Indexed Citations and Daily <1946 to May 29, 2019>

Search Strategy:

--------------------------------------------------------------------------------

1 exp infant/ or exp child/ or adolescent/ (3408806)

2 child health/ or infant health/ or adolescent health/ (3063)

3 exp child health services/ or adolescent health services/ (27488)

4 exp pediatrics/ or exp pediatricians/ or exp nurses, pediatric/ or pediatric nurse practitioners/ or pediatric assistants/ or adolescent medicine/ or hospitals, pediatric/ or exp pediatric nursing/ or exp intensive care units, pediatric/ or intensive care, neonatal/ (105479)

5 p?ediatric*.ti,ab,kf. (333122)

6 (infant* or infancy).ti,ab,kf. (466528)

7 (baby* or babies).ti,ab,kf. (69068)

8 (neonat* or newborn* or new-born*).ti,ab,kf. (379852)

9 (child* or kid or kids).ti,ab,kf. (1350497)

10 (schoolchild* or school age* or schoolage* or primary school* or elementary school* or secondary school* or high school* or highschool*).ti,ab,kf. (85399)

11 (preschool or pre-school or toddler or kindergar* or nursery).ti,ab,kf. (44102)

12 (adoles* or teen* or youth or youths or young people or young person* or young adult* or pre-teen* or preteen*).ti,ab,kf. (413570)

13 (boy* or girl*).ti,ab,kf. (226119)

14 or/1-13 (4131652)

15 exp emergency medicine/ or emergency nursing/ or emergency medical services/ or exp emergency service, hospital/ or emergency services, psychiatric/ (123706)

16 ((emergenc* or trauma) adj2 (unit or service* or department* or ward* or hospital* or room* or center* or centre*)).ti,ab,kf. (135325)

17 (er or ed or "accident and emergency" or "accident & emergency").ti,ab,kf. (143282)

18 or/15-17 (305853)

19 14 and 18 (62484)

20 implementation science/ or diffusion of innovation/ or health plan implementation/ or information dissemination/ or health information exchange/ or capacity building/ or translational medical research/ or organizational innovation/ (70221)

21 (implementation or implementing).ti,ab,kf. (261612)

22 ((data or knowledge or information) adj2 (sharing or share or distribut* or disseminat* or translat* or exchange* or communicat*)).ti,ab,kf. (49122)

23 (innovati* adj2 (diffusi* or diffuse* or sharing or share or disseminat* or scale)).ti,ab,kf. (1447)

24 ((build* or increas*) adj2 capacit*).ti,ab,kf. (21859)

25 (research adj2 (translat* or uptake or transfer* or disseminat*)).ti,ab,kf. (15084)

26 ("scale up" or "scaling up" or (strateg* adj2 (scale or scaling))).ti,ab,kf. (14045)

27 or/20-26 (401885)

28 19 and 27 (2548)

29 limit 28 to english (2432)

***************************
